# Effects of Clear-Fell Harvest on Bat Home Range

**DOI:** 10.1371/journal.pone.0086163

**Published:** 2014-01-22

**Authors:** Kerry M. Borkin, Stuart Parsons

**Affiliations:** School of Biological Sciences, University of Auckland, Auckland, New Zealand; Università degli Studi di Napoli Federico II, Italy

## Abstract

We investigated effects of roost loss due to clear-fell harvest on bat home range. The study took place in plantation forest, inhabited by the New Zealand long-tailed bat (*Chalinolobus tuberculatus*), in which trees are harvested between the ages 26–32 years. We determined home ranges by radiotracking different bats in areas that had and had not been recently clear-fell harvested. Home ranges were smaller in areas that had been harvested. Adult male bats selected 20–25 year old stands within home ranges before and after harvest. Males selected edges with open unplanted areas when harvest had not occurred but no longer selected these at proportions greater than their availability post harvest, probably because they were then readily available. This is the first radiotracking study to demonstrate a change in home range size and selection concomitant with felling of large areas of plantation forest, and thus quantify negative effects of forestry operations on this speciose group. The use of smaller home ranges post-harvest may reflect smaller colony sizes and lower roost availability, both of which may increase isolation of colonies and vulnerability to local extinction.

## Introduction

Clear-fell harvest – the logging of usually large areas of even-aged trees at regular intervals – has been heavily criticised for its potential impacts on vegetation structure and forest-dependent fauna [1]. When bats choose home ranges that include plantation forests they are likely to come into contact with harvest operations [2], [3]. However, effects on foraging activities have not been investigated for many species and most studies have taken place in areas where harvest had not recently taken place [4]. When roost numbers are reduced due to harvest operations [5], and the remaining roosts are located far from optimal foraging sites, home ranges may become larger out of necessity [6]. Conversely, as colony sizes reduce concomitant with harvest operations [5], home ranges may reduce in area. Meta-analyses of foraging studies show no consistent pattern of habitat use by bats, and therefore bat biologists are generally unable to make recommendations to forest managers about harvest prescriptions that take into account foraging requirements of individual species [7]. Consequently, well-designed radiotracking studies are required in actively managed plantation forests to investigate individual bat species' home range characteristics and habitat selection to resolve effects of harvest operations [4], [8]. We considered that for harvest to be recent it would have occurred within one year prior to the study taking place, so that effects of harvest operations may still be able to be observed. A search of the literature suggests that there have been no studies comparing bats' home ranges in areas where clear-fell harvest operations had and had not occurred recently. Consequently, we aimed to investigate the effect of clear-fell harvest operations on bat home range and habitat selection for the first time.

*Chalinolobus tuberculatus* are considered nationally vulnerable to extinction in the short-term [9] and are declining in number in each habitat type where their survival has been monitored [5], [10], [11]. They are present in plantation forests throughout New Zealand so must come into contact with clear-fell harvest operations at least occasionally [3]. However, the impact of such forest management on their habitat use is unknown. Within plantation forest long-tailed bats select home ranges that have higher proportions of near-harvest age stands and within these home ranges choose roosts in stands closest to harvest age [12], [13]. Colonies of *C. tuberculatus* roosting in mature *Pinus radiata* adjacent to recently clear-felled areas have significantly fewer bats than those where harvest has not recently occurred [5]. It is suspected that this is due to bats either being killed during harvest operations or moving to other roosting areas. Consequently, it is likely clear-fell harvest operations results in altered home range and habitat selection. *Chalinolobus tuberculatus* appear to be faithful to specific roosting and foraging areas over several years in both native and plantation forests [14], [15] so we expected that clear-fell harvest operations would also force changes in home range selection.

Home ranges in areas where harvest had recently occurred (post-harvest, P. H.) were predicted to be smaller than those of bats radiotracked in areas where harvest had not recently occurred (no harvest, N. H.) due to the felling of older stands, where they are most often detected and roost [12], [15]. Home ranges were also predicted to be smaller after harvest operations due to the smaller colony sizes found in stands adjacent to recently harvested areas [5] because *C. tuberculatus* are an example of a refuging species. Refuging species radiate out from a central communal place – their roost – to their individual foraging areas [16]. In the presence of fewer individuals refuging species have smaller home ranges [16]. To test these predictions radio-telemetry was used to investigate *C. tuberculatus* home range size within plantation forest in areas where clear-fell harvest operations had and had not recently occurred.

## Materials and Methods

*Chalinolobus tuberculatus* were captured and radiotracked over three summers (October 2006– March 2007; November 2007– March 2008; and November 2008– March 2009) within Kinleith Forest, a privately owned plantation forest. Kinleith Forest is an intensively managed exotic plantation forest, located in the Central North Island, New Zealand (37° 47′ S, 175° 53′ E). Plantings comprise mainly *Pinus radiata* with smaller *Pseudotsuga menziesii*, and *Eucalyptus* plantings. *Pinus radiata* are managed with clear-fell harvest on a 26–32 year rotation [17].

Bats were captured, handled, and radiotracked under permit from Department of Conservation (Low Impact, Research and Collection Permit BP-18899-RES under Section 53, Wildlife Act 1953) and University of Auckland Animal Ethics Committee (AEC 08/2004/R282). Bats were captured using either three-tier mistnets placed across forest roads, or harp traps or hand nets as bats emerged from roosts. Each bat was measured, weighed, and their sex and stage in the reproductive cycle was recorded. Females' abdomens were palpated to determine pregnancy. If at least one nipple was elongated with a bare area of skin surrounded it, bats were considered lactating [18]. Juvenile bats born during the summer field season (so had never bred) were identified by a lack of fusion of the phalangeal epiphyses [19]. The phalangeal epiphyses are fully fused at approximately three months of age [20]. If these joints were fused, bats were considered adult. Females were considered to have bred previously if their nipples were conspicuous [18], [20]. All captured bats were ringed on the forearm using an individually numbered 2.8 mm bat ring (The Mammal Society, United Kingdom).

Transmitters (Model BD-2, Holohil Systems Ltd, Canada) were attached to bats just behind the shoulder blades using ADOS F2 contact adhesive (CRC Industries New Zealand) after a small area of hair was clipped to ensure maximum adhesion. Transmitters weighed 0.48 g and mean transmitter load was 4.71% (range = 3.56–5.65%) of body mass. Bats were released the same night and in the same location as they were captured. From this time on they were radio-tracked continuously each night until either their signal was lost or they were stationary for over an hour without signal fluctuation. Bats were radio-tracked using a Yagi aerial and Telonics receiver (Telonics, Inc, Arizona, United States of America). A relative measure of signal strength (strong, medium, or weak), direction (measured by a compass bearing) and estimated location were all recorded. Bats were considered stationary if signal strength did not fluctuate and the compass bearing did not change. They were considered moving if signal strength fluctuated and the compass bearing changed. Locations of bats were determined by signal strength, compass bearing, observer experience, and knowledge of the area and, when possible, by bisecting or triangulating the signal direction. Accuracy of locations was estimated to be ±50 m. A very close approach was rarely possible as bats often left areas when vehicle lights were present. When possible actual locations of bats were confirmed using a “close approach” (*i.e.*, usually the bats approaching the personnel) combining simultaneous radio-telemetry and location of the bat (and identification of it's activity) using bat detectors. When compass bearings to bats were uncertain they were not recorded.

Home range characteristics were calculated using Ranges6 v1.217 (Anatrack Ltd, Wareham, United Kingdom) as described by Borkin and Parsons [13]. Home range was defined as the restricted area within which a bat moves when performing its normal activities [21]. We used the Minimum Convex Polygon (MCP) technique to determine home range size. Home range span was defined as the furthest distance from one edge of the 100% MCP home range to the other. The MCP technique of determining home range was used as it is considered relatively unaffected by the effects of autocorrelation [21]. For highly mobile species the statistical ‘time to independence’ is likely to be an overestimate of an appropriate sampling interval [21] so the interval of 15 min was systematically chosen for fixes used in analyses; the time taken for the animal to travel between the two most widely separated points within its range at the highest speed that it can attain [22]. To determine habitat selection at the landscape and local scale habitat preference was analysed at two levels, as recommended by Aebischer et al [23]. To investigate landscape-level habitat selection (selection of the home range area), the selection of habitat within 100% MCPs was compared to the available habitat within the entire study area. The entire study area was defined as the area which included all the home ranges of captured bats. All habitat types within this area were considered available to bats. Ranges8 v2.2 (Anatrack Ltd, Wareham, United Kingdom) was used to calculate both the proportion each habitat category comprised within each bat's 100% MCP and within the entire study area. To investigate local scale habitat selection (site selection), habitat preference within the 100% MCP was investigated by calculating habitat at locations and comparing this with habitat available within the home range [24] using Ranges8 v2.2. Rasters used as habitat information in habitat selection analyses were created in ArcGIS 9 (ESRI, 380 New York Street, Redlands, CA 92373-8100, USA) using data provided by the forest managers (Hancock Forest Management and Carter Holt Harvey Forests). An October ‘snapshot’ of data was chosen corresponding with the start of the summer field season, and this can be considered representative of the area for the entire summer. Changes in habitat availability due to harvest operations are relatively small over summer because of holidays taken by most forest contractors. This low rate of change throughout the plantation over the summer field season meets the required assumption that availability of habitat is constant for each radiotracking session [25]. Capture of bats did not occur in areas where harvest operations were occurring (due to health and safety concerns), so any errors in habitat classification due to harvest operations will be greatest for comparisons with the entire study area, although even these will be small, because the area harvested each year is small compared to that of the entire forest. Over the entire study period only 4.3% of the entire harvestable area of Kinleith Forest was harvested (2006 0.1%; 2007 1.4%; 2008 1.5%; 2009 1.3%; R. Black, Hancock Forest Management, Pers. Comm. 10 September 2013). We produced raster maps with age categories of planted tree species (we combined species into one unit as these were mainly *P. radiata*, with only small areas of *Eucalyptus* spp., and *Ps. menziesii*) as well as unplanted areas. Age classes were categorised as 0–5 years; 5–10 years; 10–15 years; 15–20 years; 20–25 years; 25–30 years; 30–35 years; 35–40 years; and 40–80 years in 2006 (or 83 years or 84 years in 2007 and 2008, respectively). The category ‘open unplanted areas’ included recently harvested and still unplanted stands, open areas unable to be planted (such as airstrips), native regenerating or reserve areas, and areas of pasture, mainly used for dairy farming [26].

We repeatedly radiotracked two individual bats before and after harvest operations, and during different seasons and whilst in different reproductive states. Additional methods, data, and discussion regarding these two individual bats are included in the Results S1.

A buffer of 75.0 m at bats' locations was chosen during analyses to provide an average value around locations in raster maps [24]. This large buffer was chosen to avoid potential incorrect classifications of individual raster cells; the general area is more likely to be representative of the actual raster value if an appropriate buffer radius is chosen [24]. Raster resolution matched buffer size.

Habitat selection was assessed following Neu et al. [27] using a χ^2^ goodness-of-fit test with Bonferroni simultaneous confidence intervals. Habitat selection analyses were carried out with Resource Selection for Windows 1.0 (Frank Leban ©). Individual bats were used as sample units.

We report 100% MCPs because these are the most commonly used metric used in reporting of other studies of *C. tuberculatus'* home range [28], [29] and so allow other researchers to compare those home ranges found in this study with those elsewhere (although one researcher used 95% MCPs [30]). Both the 100% and 95% MCPs are reported because their values differ (Kendall's *W* = 0.95, χ^2^(1)  = 19.0, *P*<0.001). 85% MCPs were considered core areas of bat home ranges as they were areas of particularly high home range use and were determined as in Borkin and Parsons [13]. For analyses, male and female juvenile bats were considered as the same reproductive state (*i.e.*, juvenile/never bred) as there were no differences in home range characteristics between individuals [12].

Effect sizes were calculated using Pearson's product-moment correlation coefficient, *r* [31]. Cohen's guidelines for what constitutes a small or large effect on a population were used, so a medium effect size (*r*≈0.3) represents an effect which is likely to be visible to a careful observer's naked eye (large effect size is equivalent to r≥0.5; a small effect size r≈0.1, [32]).

Whilst attempts were made to capture bats throughout the study area, this was not always possible due to the locations of colonies, and the distribution of practical capture sites. Inferences from this study should, therefore, be kept conservative.

Non-parametric Mann-Whitney tests were used to compare home range sizes and spans between N. H. and P. H. bats. Bats were considered ‘N. H.’ if no harvest operations had taken place in the forest stands adjacent to their home range for at least one year when radiotracking took place. Bats were considered ‘P. H.’ if radiotracking took place within the year after a harvest operation had taken place in the stands adjacent to their home range. Different bats were radiotracked in N. H. and P. H. groups.

## Results

Twenty individual bats were radiotracked for a median of 4.5 nights (*IQR* = 3.3–6.0, minimum = 2, maximum = 15), and a median 52.5 fixes (*IQR* = 27.5–99.0, minimum = 7, maximum = 180) were collected from each bat. These twenty bats represented a range of age and sex classes and were radiotracked either N. H. or P. H. (Table 1).

**Table 1:** Long-tailed bats radiotracked in areas that had (P. H.) and had not (N. H.) been recently clear-fell harvested.

Capture period	Reproductive state					Total
	Juvenile	Juvenile	Adult Male	Pregnant Female	Lactating Female	
	Male	Female				
N. H.	1	3	5	0	1	10
P. H.	1	0	4	3	2	10
Total	2	3	9	3	3	20

There was no difference in the number of nights that bats in different reproductive states were radiotracked (*H* (3)  = 2.16, *P* = 0.572) and there was no difference in the number of fixes obtained for bats radiotracked in different reproductive states (*H* (3)  = 0.85, *P* = 0.859). There were no statistical differences in home range sizes or spans between bats in different reproductive states (100% MCP: *H* (3)  = 4.83, *P* = 0.188; 95% MCP: *H* (3)  = 3.00, *P* = 0.418; 85% MCP: *H* (3)  = 1.78, *P* = 0.653; Range span: *H* (3)  = 3.34, *P* = 0.365). There were no linear relationships between number of fixes collected and 100%, 95% or 85% MCPs or range span (100% MCP: *r* = −0.04, *P* (1-tailed)  = 0.441; 95% MCP: *r* = −0.20, *P* (1-tailed)  = 0.203; 85% MCP: *r* = −0.22, *P* (1-tailed)  = 0.176; Range span: *r* = 0.03, *P* (1-tailed)  = 0.456). There were no linear relationships between the number of nights bats were radiotracked and 100%, 95% or 85% MCPs or range span (100% MCP: *r* = 0.10, *P* (1-tailed)  = 0.344; 95% MCP: *r* = −0.08, *P* (1-tailed)  = 0.365; 85% MCP: *r* = −0.20, *P* (1-tailed)  = 0.198; Range span: *r* = 0.11, *P* (2-tailed)  = 0.328). Finally, there was no difference in the number of nights that pre- and post-harvest bats were radiotracked (*U* = 42.50, *Z* = −0.58, *P* (2-tailed)  = 0.59, *r* = −0.13). The only individual bat that did not approach asymptotes for their home ranges were a juvenile female (JF9329) who had the largest home range size and span and for whom only seven locations were obtained. The inability to obtain asymptotes for individuals even when large amounts of data is collected is common when radiotracking individuals that have biological reasons for increasing home range sizes [21]. Due to this bat's specific explanation for increasing home range size it was included in analyses. Data integrity was therefore considered acceptable for further analyses.

### Effect of harvest operations on home range characteristics

As home range sizes and spans of bats in different reproductive states did not differ home range data were pooled for comparisons of N. H. and P. H. home ranges. Home range sizes and range span of P. H. bats were all smaller than those of N. H. bats (100% MCP: N. H.  = 792.6 ha (*IQR* = 527.2–1635.0), P. H.  = 114.7 ha (*IQR* = 53.2–246.2) *U* = 15, *P* (1-tailed)  = 0.007, *r* = −0.59; 95% MCP: N. H.  = 542.6 ha (*IQR* = 425.0–839.8), P. H.  = 79.0 ha (*IQR* = 30.8–239.1) *U* = 13, *P* (1-tailed)  = 0.002, *r* = −0.63; 85% MCP: N. H.  = 454.8 ha (*IQR* = 233.5–536.5), P. H.  = 49.6 ha (*IQR* = 21.3–82.1) *U* = 16, *P* (1-tailed)  = 0.004, *r* = −0.57; Range span: N. H.  = 5609.5 m (*IQR* = 4578.1–7208.1), P. H.  = 2085.9 m (*IQR* = 1177.5–3620.9)*U* = 8, *P* (1-tailed) <0.001, *r* = −0.71).

**Figure 1:**
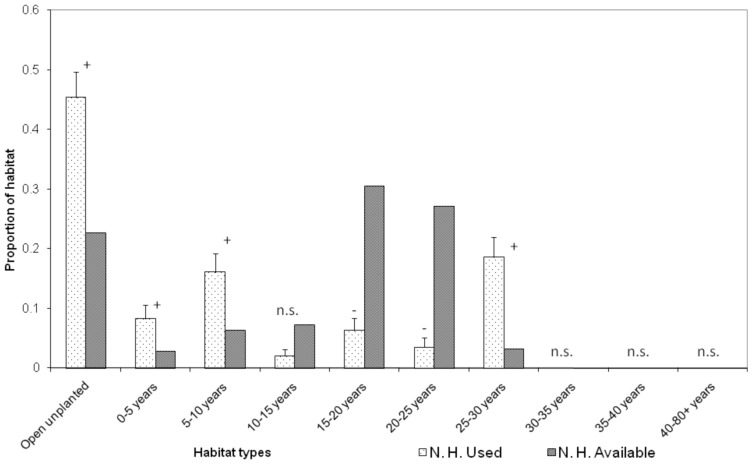
Habitat use versus availability within adult male long-tailed bat home ranges pre-harvest (*n* = 5). Habitat availability is calculated as the habitat composition of the entire study area. Age classes include planted trees of *Pinus radiata*, *Pseudotsuga menziesii* and *Eucalyptus* spp. The proportion of each habitat that is used is expressed as the proportion of night-time locations bats were radiotracked to. Symbols +/−/n.s. indicate whether habitat types were selected/avoided/used in proportion to their availability, respectively.

**Figure 2:**
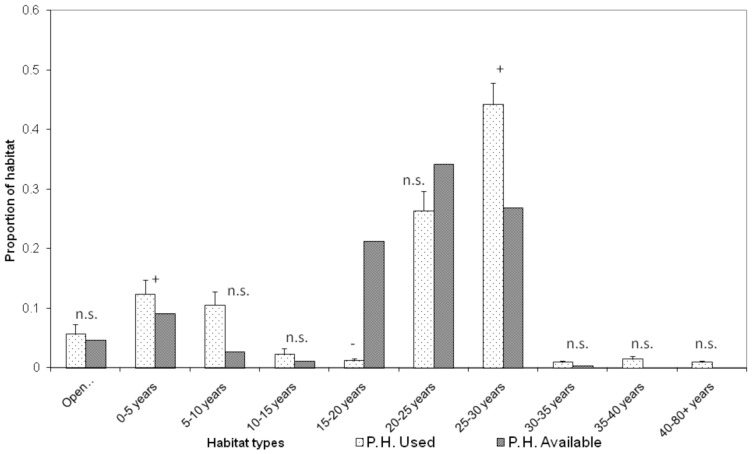
Habitat use versus availability within adult male long-tailed bat home ranges post-harvest (*n* = 4). Habitat availability is calculated as the habitat composition of the entire study area. Age classes include planted trees of *Pinus radiata*, *Pseudotsuga menziesii* and *Eucalyptus* spp. The proportion of each habitat that is used is expressed as the proportion of night-time locations bats were radiotracked to. Symbols +/−/n.s. indicate whether habitat types were selected/avoided/used in proportion to their availability, respectively. Open unplanted habitat is abbreviated as Open.

Adult male bats were the only reproductive class to be radiotracked in comparable numbers N.H. and P.H. so we only compared these during analyses of habitat selection. Adult male bats chose sites within their home ranges non-randomly both N. H. and P. H. operations (N. H.: G _adj_  = 282.45, *d.f.*  = 9, *n* = 5, *P*<0.0001; P. H.: G _adj_  = 173.82, *d.f.*  = 9, *n* = 4, *P*<0.0001, Fig. 1, Fig. 2). N. H. males selected sites within open unplanted areas, 0–5, 5–10 and 25–30 year old stands and avoided sites within 15–20 and 20–25 year old stands (*P*<0.05, Fig. 1). P. H. males selected sites within 5–10 and 25–30 year old stands and avoided sites within 15–20 year old stands (*P*<0.05, Fig. 2).

## Discussion

Our results suggest a pattern of smaller home ranges after clear-fell harvest operations. These results are interesting, particularly as similar studies of the effects of large scale habitat removal on bats appear absent from the peer-reviewed literature. Indeed, many of the studies that comment on the effect of harvest operations took place in areas where harvest operations had not occurred recently (see Miller et al. [4] for a review). Alternatively, impacts have frequently been inferred indirectly based on echolocation call rates and not use by individuals [4], [33], [34], [35], [36].

Whilst small sample sizes of this study mean that we have interpreted our results conservatively, sample sizes similar to these are common for published research into the ecology of threatened bats [37], [38], [39], [40]. In addition, when studying endangered species such as *C. tuberculatus*, smaller sample sizes may be satisfactory so that the ability to identify possible causes for concern is maintained [41].

The smaller home ranges found after clear-fell harvest operations contrast with those found after the small-scale removal of only the preferred roosts of *Thyroptera tricolor* [42]. That study took place in an area of relatively high roost availability and found *T. tricolor* increased their relatively small home range sizes and the number of tree species they used for roosting. We suggest our differing results are due to the far larger home ranges that *C. tuberculatus* exhibit [13], [28] in an area of extremely low roost availability, which further declines with harvest operations [5] due to the removal of all vegetation.

The small home ranges of bats following harvest operations are likely caused by a combination of the high proportion of edges bordering open area – areas of high invertebrate abundance [43], low roost availability [15] and reduced colony sizes that are concomitant with clear-fell harvest [5], as well as the fidelity to traditionally-held home ranges, roosting areas [15], and social groups that *C. tuberculatus* exhibit [14]. Smaller home ranges post clear-fell harvest operations may reflect the reductions that occur in colony sizes after harvest operations [5]. *Chalinolobus tuberculatus* appear to have relatively exclusive foraging areas [28] and when fewer bats are present individuals do not need to travel as far from the few available roosts to find unoccupied foraging areas [16]. Consequently, with smaller colonies it is possible to have smaller home ranges. When home ranges are food resource rich – in the case of *C. tuberculatus* when they contain areas of high invertebrate abundance – it is likely that home range size can also be reduced. This has already been noted in a variety of mammal species including bears [44]; lemurs [45]; and coyote [46] as well as bats [47]. After the initial home range contraction, movement into new areas may be slow because it is likely bats are a refuging species (central place foragers [16], [48]) with limited ability to explore new areas due to their need to maintain food intake and use the few remaining known roosts.

N. H. adult male bats selected edges of open unplanted areas within their home ranges. In contrast, P. H. males no longer selected these areas, probably because they were highly available post-harvest operations. We suggest that these bats select the areas where open unplanted areas meet older stands – the edges, and not the open unplanted areas themselves – because *C. tuberculatus* rarely cross open areas [30] and generally travel along linear landscape features [49].

We suggest that selection of 25–30 year old stands within home ranges of adult males both P. H. and N. H. indicate bats spend large amounts of time near the oldest available trees where they are most likely to roost [15], [50] and where overnight temperatures and wind speeds are most effectively buffered [51], [52].

This study provides some of the first evidence that bat home range and habitat selection are affected by clear-fell harvest. The effect of a small, contracted, home range on a individual bat may increase isolation of populations within preferred areas and the likelihood of local extinction [53]. We expect the small home ranges found in this study, combined with the loss of roosts during harvest and smaller colony sizes post-harvest, indicate that populations of bats within forests that are regularly harvested may be placed under pressures that are not present in other habitat types [5]. Bat populations are already under pressure from predation [11], roost loss [54], disturbance of roosts by humans and competition with introduced species for roost sites [55]. This study took place in a plantation forest that is adjacent to large areas of native forest with bat populations that may act as a source. Whether bats are able to sustain populations long-term without nearby source populations when under the additional pressures associated with harvest operations is unclear. We suggest that the next step in investigating effects of clear-fell harvest on individuals should involve long-term mark-recapture studies in areas where harvest occurs so that condition, reproductive success, and survival can be monitored.

## Supporting Information

Figure S1 **Adult female 2859 home ranges overlap whilst lactating in February and pregnant then lactating during November 2007.** Home ranges are displayed over a raster of unplanted areas and age classes of planted and harvested areas. Note her use of space changed between summers coinciding with the harvest of a stand in the bottom right of her February 2007 home range. This stand was harvested during winter 2007 prior to her November 2007 radiotracking session. She no longer used this area in November 2007.
(TIF)

Figure S2 **Adult female 2860 home ranges overlap whilst lactating in February and pregnant during November 2007.** Home ranges are displayed over a raster of unplanted areas and age classes of planted and harvested areas. Note her use of space changed between summers coinciding with the harvest of a stand in the bottom right of her February 2007 home range. This stand was harvested during winter 2007 prior to her November radiotracking session. She no longer used this area in November 2007.
(TIF)

Results S1 (DOC)
