# Supplementary figures and images for: Effects of Clear-Fell Harvest on Bat Home Range
Source: PLoS One. 2014 Jan 22;9(1):e86163. doi: 10.1371/journal.pone.0086163 (PMC3899175; doi:10.1371/journal.pone.0086163)

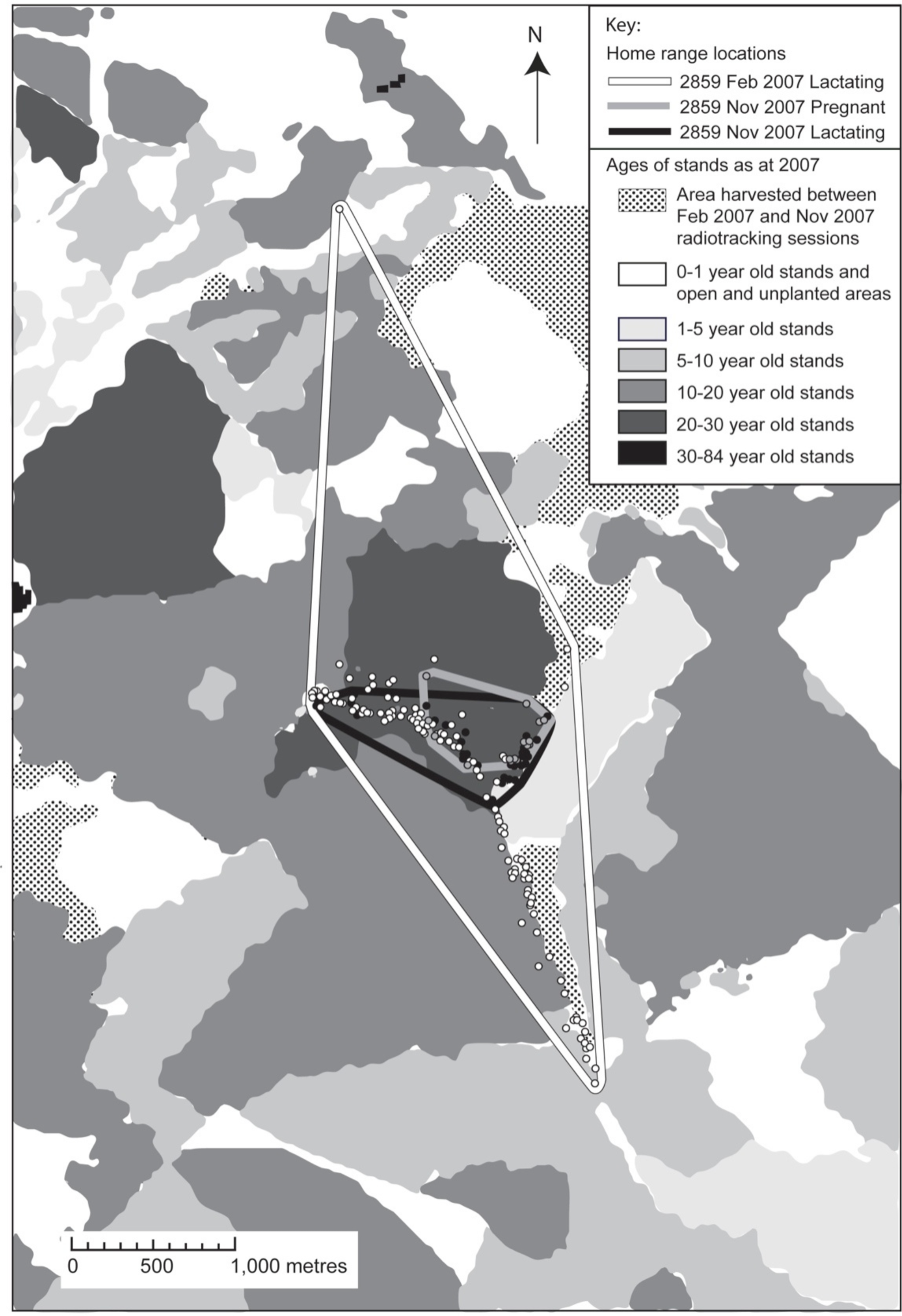

Supplement: Figure S1 — Adult female 2859 home ranges overlap whilst lactating in February and pregnant then lactating during November 2007. Home ranges are displayed over a raster of unplanted areas and age classes of planted and harvested areas. Note her use of space changed between summers coinciding with the harvest of a stand in the bottom right of her February 2007 home range. This stand was harvested during winter 2007 prior to her November 2007 radiotracking session. She no longer used this area in November 2007. (TIF) [file pone.0086163.s001.tif]

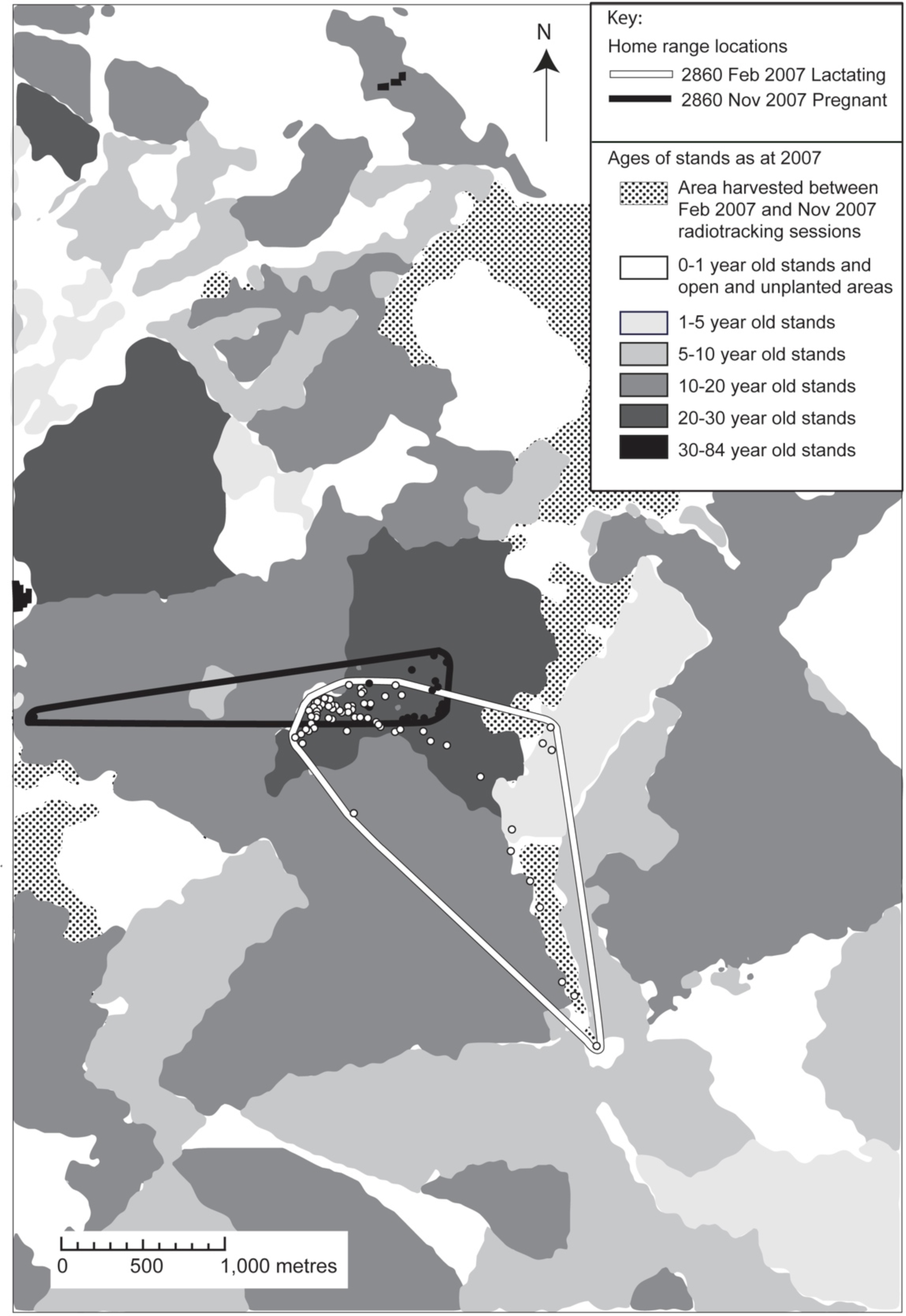

Supplement: Figure S2 — Adult female 2860 home ranges overlap whilst lactating in February and pregnant during November 2007. Home ranges are displayed over a raster of unplanted areas and age classes of planted and harvested areas. Note her use of space changed between summers coinciding with the harvest of a stand in the bottom right of her February 2007 home range. This stand was harvested during winter 2007 prior to her November radiotracking session. She no longer used this area in November 2007. (TIF) [file pone.0086163.s002.tif]
