# Supplementary material for: Effects of Clear-Fell Harvest on Bat Home Range
Source: PLoS One. 2014 Jan 22;9(1):e86163. doi: 10.1371/journal.pone.0086163 (PMC3899175; doi:10.1371/journal.pone.0086163)
Supplement: Results S1 — (DOC) [file pone.0086163.s003.doc]

**Methods**

We also used Ranges6 v1.217 to determine overlap matrices between 100% MCP home ranges of individual bats radiotracked in different seasons and reproductive states as outlined by Kenward et al. [1].

Of the captured bats, 2 individual females were repeatedly radiotracked (AF2860 and AF2859) during different field seasons and reproductive states so that the total number of home ranges identified was 23. The repeatedly radiotracked bats were only included once in comparative measures to avoid pseudoreplication, and were included whilst pregnant.

When newly lactating bat (AF2859) that had given birth the night prior to radiotracking did not reach an asymptote. The newly lactating bat’s data was only used in the analysis of overlap between bats radiotracked in two summers, as this bat was radiotracked repeatedly.

**Results**

Home range overlap between different summers and reproductive states

Two adult female bats (AF2859, and AF2860) were captured and radiotracked in both the 2006-2007 and 2007-2008 field seasons. Home ranges of both bats overlapped between summers and when in different reproductive conditions. Harvest operations had occurred in the area immediately adjacent to both bats’ home ranges between radiotracking sessions.

Both bats were captured when lactating in February 2007 and then again when pregnant in November 2007. Whilst lactating AF2859’s 100% MCP was 477.1 ha and it’s range span 5551 m (Fig.1). The next summer, whilst pregnant, its 100% MCP was 59.43 ha and its range span 1417 m (Fig.1). The average home range overlap for AF2859 between February and November was 56.2%. AF2859’s November home range was overlapped 100.0% by its February home range, but only 12.4% of AF2859’s February home range was overlapped by its November home range.

Whilst lactating in February AF2860’s 100% MCP was 212.2 ha and it’s range span 2709 m (Fig.2). The next summer, whilst pregnant, its 100% MCP was 32.2 ha and its range span 1081 m (Fig.2). The average home range overlap for AF2860 between February and November was 19.6%. AF2860’s November home range was overlapped 30.1% by its February home range, but its February home range was only overlapped 9.1% by its November home range.

Fig. S1.

Fig. S2.

Home range overlap when bats were in different reproductive states within the same summer

One adult female (AF2859) was radiotracked whilst pregnant in November 2007, and then again when newly-lactating. She was radiotracked prior to parturition for 10 nights and after parturition for 4 nights. Whilst pregnant her 100% MCP was 59.4 ha and range span 1417 m. When lactating her 100% MCP was 65.5 ha with a range span of 2551 m. The home ranges of AF2859 overlapped when she was in different reproductive conditions within the same summer (Fig.1). Her average home range overlap when pregnant and lactating was 65.2%. Her pregnant home range overlapped 84.4% of her lactating home range. Her lactating home range overlapped 45.9% of her pregnant home range.

**Discussion**

The few bats able to be radiotracked repeatedly showed fidelity to traditional home ranges between summers. Home ranges of the repeatedly radiotracked individuals overlapped before and after harvest and in both cases were smaller after harvest.

Home range overlap may have been higher between seasons if the harvest of a *P. radiata* stand frequently used by bats in the summer of 2006-2007 had not occurred. As harvest of forest stands occurs within home ranges, home ranges move as demonstrated by the two repeatedly radiotracked bats in this study. Bats may also initially contract their home ranges at the time of harvest to areas they know well. It appears that this is the case for the few bats able to repeatedly radiotracked in Kinleith Forest and is supported by the finding that home ranges are smaller post-harvest operations. If alternative stands suitable for roosting are spaced within the home range size of bats, they may know of potential roosts there, minimising search times for new roosts and reducing impacts of harvest operations.

**References**

1. Kenward RE, South AB, Walls SS (2003) Ranges6 v1.2 : For the analysis of tracking and location data.: Anatrack Ltd. Wareham, United Kingdom.

2. Daniel S, Korine C, Pinshow B (2008) Central-place foraging in nursing, arthropod-gleaning bats. Canadian Journal of Zoology 86: 623 - 626.

3. Hamilton WJ, Watt KEF (1970) Refuging. Annual Review of Ecology and Systematics 1: 263 - 286.

Figure 1. Adult female 2859 home ranges overlap whilst lactating in February and pregnant then lactating during November 2007. Home ranges are displayed over a raster of unplanted areas and age classes of planted and harvested areas. Note her use of space changed between summers coinciding with the harvest of a stand in the bottom right of her February 2007 home range. This stand was harvested during winter 2007 prior to her November 2007 radiotracking session. She no longer used this area in November 2007.

Figure 2. Adult female 2860 home ranges overlap whilst lactating in February and pregnant during November 2007. Home ranges are displayed over a raster of unplanted areas and age classes of planted and harvested areas. Note her use of space changed between summers coinciding with the harvest of a stand in the bottom right of her February 2007 home range. This stand was harvested during winter 2007 prior to her November radiotracking session. She no longer used this area in November 2007.
